# A difficult case of juvenile dermatomyositis complicated by thrombotic microangiopathy and purtscher-like retinopathy

**DOI:** 10.1186/1546-0096-12-S1-P275

**Published:** 2014-09-17

**Authors:** Federica Vanoni, Catherine Jorgensen, Paloma Parvex, Carlo Chizzolini, Michaël Hofer

**Affiliations:** 1 Unité Romande de Rhumatologie Pédiatrique (URRP), CHUV, Lausanne, Switzerland; 2 Département de l'enfant et de l'adolescent Hôpital des Enfants - Hôpitaux Universitaires de Genève, HUG, Geneva, Switzerland; 3 Spécialités de Médecine Serv Immunologie Allergologie , Hôpitax Universitaires Genève, Genève, Switzerland; 4 Unité Romande de Rhumatologie Pédiatrique (URRP)épartement Médico - Chirurgical de Pédiatrie (DMCP), Centre Hospitalier Universitaire Vaudois CHUV, Lausanne, Switzerland; 5 TBC

## Introduction

Juvenile dermatomyositis (JDM) is a multisystem disease of uncertain origin resulting in chronic muscle and skin inflammation. Disease’s complications are calcinosis and cutaneous ulcerations, lipodystrophy, joint contractures, interstitial lung disease, cardiac involvement, digestive and central nervous system vasculitis.

## Objectives

We report here a case of severe JDM complicated by thrombotic microangiopathy (TMA).

## Methods

Case report.

## Results

A 16 year old girl was admitted for fever, diffuse pain, asthenia, sore throat and generalised papular rash. Initial work-up showed leukopenia, elevation in creatine phosphokinase (CK) (650 U/l) and transaminase. Epstein-Barr Virus (EBV) serology was compatible with acute infection and the initial treatment was symptomatic.

Subsequent deterioration of general conditions, progressive polymyositis with regular increase in muscle enzymes (CK up to 39000 U/l), massive muscle swelling and maculo-papular rash were consistent with a diagnosis of JDM. MRI confirmed muscular inflammatory involvement.

Sudden onset of blurred vision, haemolytic anaemia (haemoglobin 53 g/l and schistocytes) and thromobocytopenia (21 G/l) lead to further investigation. Complete work-up showed Purtscher-like retinopathy, renal failure (creatinine 150 umol/l) and pancreatitis (lipase 570 U/l).

Renal and Muscle biopsy showed microangiopathy with capillary endothelium necrosis and mild inflammation consistent with JDM and TMA. Dosing of ADAMST-13 activity was normal.

Patient failed to respond to pulse therapy with methylprednisolone, intravenous immunoglobulin, plaquenil, rituximab and cyclophosphamide. Patient showed also partial response to plasma exchange therapy, with successive deterioration of clinical conditions and biological parameters.

Treatment with Eculizumab, a monoclonal antibody against the C5 protein fraction of complement system, 900 mg once/week for 5 weeks and then 1200 mg once 2/week was effective in improving clinical condition and biological parameters.

## Conclusion

### Conclusion

This report emphasizes that early recognition of TMA and prompt treatment are important in children with severe JDM associated with anaemia and thrombocytopenia. Eculizumab is to consider when plasma exchange is not effective enough.

## Disclosure of interest

None declared.

